# GIFT-Cloud: A data sharing and collaboration platform for medical imaging research

**DOI:** 10.1016/j.cmpb.2016.11.004

**Published:** 2017-02

**Authors:** Tom Doel, Dzhoshkun I. Shakir, Rosalind Pratt, Michael Aertsen, James Moggridge, Erwin Bellon, Anna L. David, Jan Deprest, Tom Vercauteren, Sébastien Ourselin

**Affiliations:** a Translational Imaging Group, Centre for Medical Imaging Computing, University College London, London, UK; b Institute for Women's Health, University College London, London, UK; c Department of Imaging & Pathology, UZ Leuven, Leuven, Belgium; d Department of Medical Physics, UCL Hospitals, London, UK; e Department of Information Technology, UZ Leuven, Leuven, Belgium; f Department of Obstetrics, UZ Leuven, Leuven, Belgium

**Keywords:** Data sharing, Biomedical research, Cross-disciplinary research, Anonymisation, Deidentification, Fetal surgery

## Abstract

**Objectives:**

Clinical imaging data are essential for developing research software for computer-aided diagnosis, treatment planning and image-guided surgery, yet existing systems are poorly suited for data sharing between healthcare and academia: research systems rarely provide an integrated approach for data exchange with clinicians; hospital systems are focused towards clinical patient care with limited access for external researchers; and safe haven environments are not well suited to algorithm development. We have established GIFT-Cloud, a data and medical image sharing platform, to meet the needs of GIFT-Surg, an international research collaboration that is developing novel imaging methods for fetal surgery. GIFT-Cloud also has general applicability to other areas of imaging research.

**Methods:**

GIFT-Cloud builds upon well-established cross-platform technologies. The Server provides secure anonymised data storage, direct web-based data access and a REST API for integrating external software. The Uploader provides automated on-site anonymisation, encryption and data upload. Gateways provide a seamless process for uploading medical data from clinical systems to the research server.

**Results:**

GIFT-Cloud has been implemented in a multi-centre study for fetal medicine research. We present a case study of placental segmentation for pre-operative surgical planning, showing how GIFT-Cloud underpins the research and integrates with the clinical workflow.

**Conclusions:**

GIFT-Cloud simplifies the transfer of imaging data from clinical to research institutions, facilitating the development and validation of medical research software and the sharing of results back to the clinical partners. GIFT-Cloud supports collaboration between multiple healthcare and research institutions while satisfying the demands of patient confidentiality, data security and data ownership.

## Highlights

• A platform for sharing medical imaging data between clinicians and researchers.
• Extensible system connects three hospitals and two universities.
• Simple for end users with low impact on hospital IT systems.
• Automated anonymisation of pixel data and metadata at the clinical site.
• Maintains subject data groupings while preserving anonymity.

## 1 Introduction

GIFT-Cloud is a secure data platform that simplifies and automates the process of accessing and sharing data for medical imaging research. GIFT-Cloud has been developed to support GIFT-Surg [1], a multi-institution collaboration between academic and clinical researchers developing novel ways to image the fetus for fetal surgery. The GIFT-Cloud platform has general applicability and could be adapted to benefit medical imaging research projects in other fields.

Medical imaging is widely used in clinical practice, with applications ranging from screening and diagnosis to treatment planning and image-guided surgery. The continuing development of novel imaging modalities, improved protocols and computer-assisted analysis software has huge potential to improve disease assessment and patient outcome. These advances require ever closer collaboration between academia, industry and healthcare providers.

One major issue in such collaborations is managing the images and associated metadata needed for developing, testing and validating novel algorithms for medical image analysis. A wide range of clinical data are required that offer a representative sample of the disease condition being studied. As research increasingly specialises on a narrower range of applications and diseases, it becomes harder to obtain sufficient clinical data from any single institution. Firstly, for rare disease conditions individual hospitals may see only a limited number of cases per year. Secondly, due to the rapid changes in imaging technology, data acquired using older hardware or protocols may be of less research value. Therefore, specialised research increasingly benefits from collaboration with multiple clinical institutions. The legal and technical requirements of data sharing are major considerations within any such projects.

Patient confidentiality and data protection are enshrined in national, international and institutional regulations [2], [3] limiting how and where patient-derived data are acquired and stored. Data use may require patient consent and permission from ethics boards, and institutions may wish to protect institutional copyright and intellectual property [4]. A significant technical challenge is the removal of personal identifiable information (anonymisation) before data leave the secure clinical environment. Naive approaches to anonymisation simply remove personal data, but this makes it impossible to later recombine data from the same subject, precluding multi-modal image analysis or follow-up studies. Further technical challenges include the sharing of data between protected secure networks, obtaining unified access to data originating from separate storage systems, data backup, fault tolerance in the case of interrupted data transfer and reconciling the need for large volumes of data with the bandwidth limitations of data transfer between institutions.

Collaborative research projects often do not consider these issues in the early project stages and this frequently results in ad-hoc and suboptimal solutions for sharing research data. Manually-driven processes are potentially prone to human error, and sharing data can quickly become a burden to clinicians, discouraging data provision beyond the minimum necessary. Conversely, the results of image processing research often do not easily flow back to the clinicians, limiting their ability to evaluate this research and therefore its clinical potential.

The goal of GIFT-Cloud is to provide a flexible, clinician- and researcher-friendly system for anonymising and sharing data across multiple institutions. GIFT-Cloud aims to satisfy the demands of patient confidentiality, data security and legal agreements for data sharing and ownership. GIFT-Cloud brings a number of benefits to the GIFT-Surg project. These include encouraging more data sharing, standardising and simplifying the data transfer and anonymisation processes, reducing the time burden on clinicians and researchers, providing centralised storage and backup of research data and simplifying the sharing of researcher-derived results to clinicians for validation.

GIFT-Cloud achieves these aims by building upon existing, well-established cross-platform technologies. GIFT-Cloud software is available for research use under a BSD Simplified (BSD-3) licence [5].

In this article we describe the GIFT-Cloud system and illustrate its use in the clinician–researcher workflow. The article is structured as follows. Section 2 reviews published systems in the field, and discusses the additional requirements that have motivated the development of GIFT-Cloud. Section 3 is an overview of the system features and technology. Section 4 presents a case study of how data are acquired and exchanged using GIFT-Cloud for an example application in the GIFT-Surg project. Section 5 presents a qualitative evaluation of the system including descriptions of features, scalability, performance and testing. Finally, Section 6 presents discussions and conclusions.

## 2 State of the art

Healthcare institutions typically store their imaging data in a Picture Archiving and Communication System (PACS) [6], which relies on the DICOM standard [7] for communicating and managing the data. While data exchange with a PACS is highly standardised, the same is not true for the overall Electronic Medical Record (EMR). Both PACS and EMR may support remote access but that is solely to support clinical routine and not research use of the data. This is because healthcare providers have obligations towards patient confidentiality and data security and are naturally reluctant to give external users access to their systems.

One potential approach which permits data sharing while addressing confidentiality issues is to create a “data safe haven”. This is a secure environment linked to a clinical institution in which research software can be run on patient-identifiable data, without giving researchers direct access to the data. To protect patient confidentiality, only aggregated data are exported, with no output for individual patients. Data safe havens are suited to using mature, stable software in clinical research. However, they are too restrictive for novel algorithm development, where individual patient results are necessary for analysing the performance and iteratively testing improvements to the algorithm.

In practise, therefore, developing novel image processing algorithms requires the research institution to host data on its own systems. This requires transfer of data from the clinical institutions to the research institution. Typically, anonymisation must be performed on the clinical site to prevent identifiable patient data from leaving the hospital. A common result is a process where clinicians manually anonymise data for storage on removable media or a shared system, after which researchers must copy the data onto a shared resource within the academic institution. Anonymisation is manually initiated by the clinician either using functionality on the data generating equipment, or from the PACS, or using standalone software. However, this is prone to patient data leakage if the software is not used with consistent settings, something that is difficult to guarantee in a manual procedure [8]. Manual exporting processes are also error-prone, for example if individual images are mistakenly exported instead of series. Anonymisation systems do not normally support anonymisation of pixel data. DicomCleaner [9] is one of the few programs supporting pixel data anonymisation, although only in an interactive mode.

Research institutions are increasingly using server-based data management systems to facilitate data sharing. Established research systems include XNAT [10], CTP [11], HID [12], COINS [13], LORIS [14], BIRN [15] and TCIA [16]. While mostly focussed on specific research areas and particular workflows, these systems often have more general extensibility. For example, XNAT was developed for neuroimaging research but is now used in other areas [15], [17]. These systems can considerably improve collaboration, but they do not always solve nor simplify the problem of obtaining and anonymising data from clinical sources. This is particularly true where the clinical and research data are hosted on different networks, requiring an intermediate connection such as the Internet or a dedicated data connection. While some research systems permit secure data upload from the hospital, they cannot in general be connected directly to the PACS or hardware devices due to risk of sending non-anonymised data outside the clinical environment and the security implications of connecting medical devices directly to the Internet. To address these concerns, an intuitive process is needed for clinicians to transfer data from the clinical to the research system while guaranteeing anonymisation and maintaining the data security of the hospital system.

These considerations motivate a “gateway” approach in which an intermediate server is installed within the clinical environment. That server typically acts as a DICOM destination allowing data to be pushed from the PACS or scanners. The received data are anonymised by the gateway and sent to the server in the research institution. The technical details of the file transfer are hidden from the end user, but will typically involve a direct data connection or Internet transfer using secure communication protocols. Internet-based transfer is more practical due to the time and cost involved in installing a direct data link. However, to meet identity protection requirements the gateway must be installed within the clinical site, be able to receive data from the PACS and provide local data anonymisation before encrypted upload to the remote server.

Integrating a gateway service solely with the hospital PACS may be insufficient for obtaining all the required data, since the PACS system may not store all the data desired by researchers. This is because some data may not be in a format suitable for storage in the PACS, or because large datasets such as endoscopic video or 3D ultrasound volumes may not be archived if they are not considered to have future diagnostic benefit, even if they have substantial value for researchers developing and validating novel methods. A general data-sharing system needs to be flexible enough to support obtaining of data from multiple different systems and devices.

None of the existing research systems support all the features required by the GIFT-Surg project, such as grouping of datasets using pseudonymised patient identifiers, maintaining a hospital-side patient list of anonymised identifiers, providing a mechanism for client-side anonymisation of pixel and metadata and upload of data for multiple subjects simultaneously. Rather than develop a new system from scratch, GIFT-Cloud builds upon the well-established XNAT system to provide these new capabilities, extending the server framework and adding custom gateway and uploading software to provide a flexible system that meet the needs of the clinicians and researchers.

## 3 Description of GIFT-Cloud

### 3.1 System overview

**Fig. 1:**
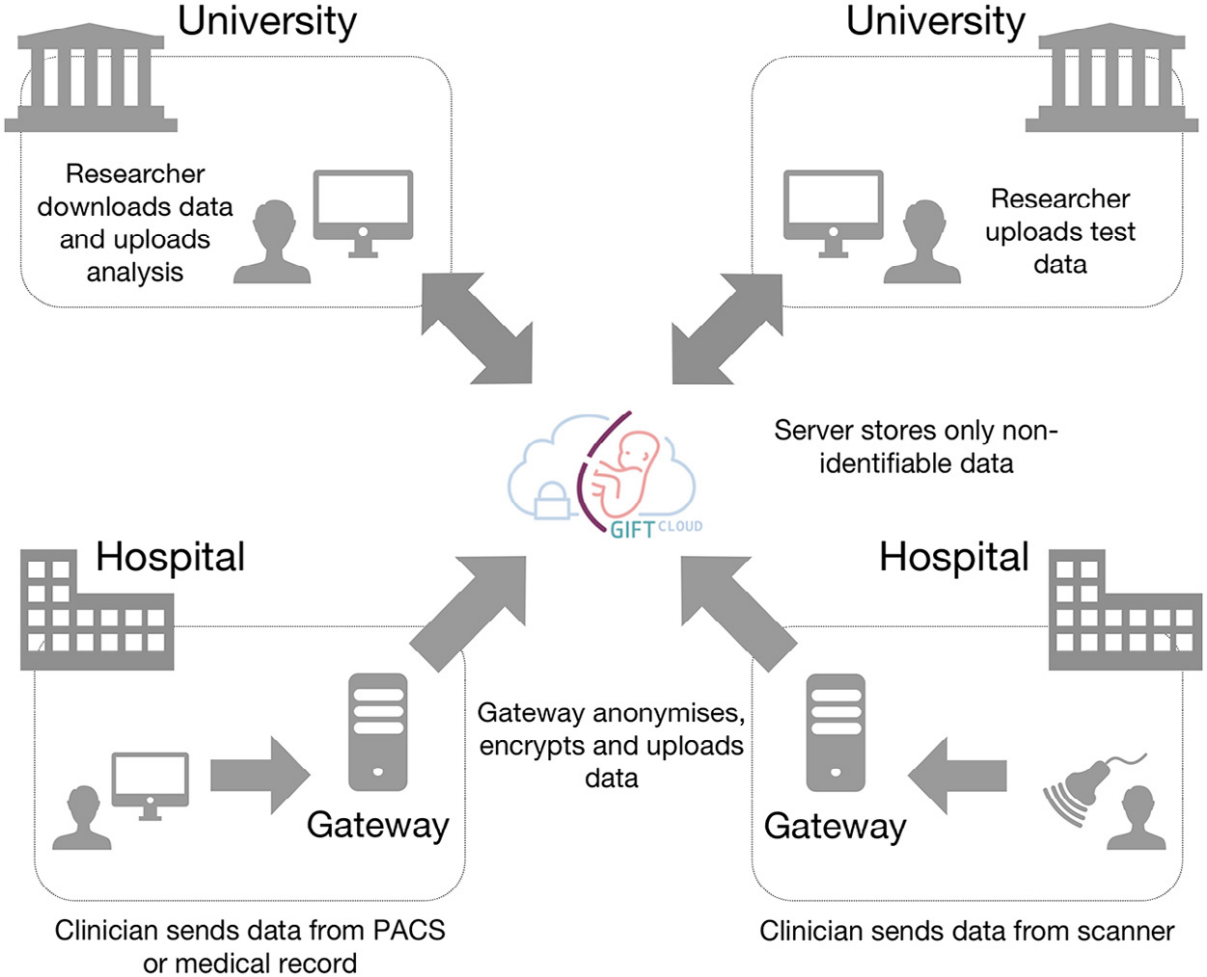
Simplified diagram illustrating collaboration using GIFT-Cloud. Data can be provided from hospitals as part of the routine workflow for patient care. Researchers can upload analysis of the data, which are then available to other collaborators. Confidential patient information never leaves the source hospital.

A simplified system diagram of GIFT-Cloud is shown in Fig. 1. GIFT-Cloud consists of a central GIFT-Cloud Server and optional GIFT-Cloud Gateway servers within each clinical institution. GIFT-Cloud is built using cross-platform technologies, offering flexibility with regard to the required hardware and operating systems. GIFT-Cloud provides data upload with fully automated, configurable, client-side anonymisation of patient information contained in both metadata and pixel data. The system provides automated subject matching and patient list mapping while preserving subject anonymity in the research data. The system requirements are summarised in Table 1.

### 3.2 GIFT-Cloud Server

Anonymised research data are hosted on a dedicated GIFT-Cloud Server running a customised version of XNAT 1.6 [10]. Users can access and download data within the collaborating institutions via a secure website extended from the XNAT web interface. A Representational State Transfer Application Programming Interface (REST API) provides a set of protocols and services that can be accessed programmatically, which allows seamless integration of the research database directly into the software applications being developed by researchers. Our GIFT-Cloud Server is a dedicated virtual machine running CentOS Linux 7.2 with the database managed using PostgreSQL 9.2 and XNAT 1.6 running under Apache Tomcat 7.0, Oracle Java 1.7 and the Apache 2.4 daemon.

### 3.3 GIFT-Cloud Uploader

Using the GIFT-Cloud Uploader software, users can interactively import data from the local file system or by querying a local PACS. GIFT-Cloud Uploader is a cross-platform Java Web Start application, custom-developed for uploading data directly by users or for running as a service on GIFT-Cloud Gateway servers. The software can be installed directly from the GIFT-Cloud website and can be updated without the need for incoming connections into the hospital's network. The Uploader makes use of open-source software including XNAT [10] (BSD Simplified), DicomCleaner [9] (BSD Simplified with additional clause) and dcm4che [18] (Mozilla Public License 1.1).

### 3.4 Integration with hospital systems

Optional Gateway servers allow each institution to provide image uploading mechanisms suited to local policies, such as sending data from the PACS or the electronic medical record. Each clinical institution can provide a Gateway system by installing the cross-platform GIFT-Cloud Uploader software on an appropriate on-site server. This server is provided by the institution in a way that conforms to their local security and maintenance requirements. Users can DICOM Push data to the Gateway from PACS, scanners and other DICOM-compatible devices; the data will automatically be anonymised by the Gateway before uploading. Non-DICOM data can be uploaded by copying to a shared folder on the Gateway server, an established mechanism that is simple to integrate with the hospital IT system while remaining secure within the hospital environment. Data upload is fault-tolerant; if uploading fails, for example due to a server outage, the file is retained for uploading at a later time. This shields the clinician and sending institution from these concerns regarding the current status of the research server.

### 3.5 Anonymisation

GIFT-Cloud automatically anonymises patient information in DICOM metadata and burnt-in annotations in pixel data. For non-DICOM data, custom anonymisation procedures are developed as required. Anonymisation is performed by the GIFT-Cloud Uploader software on the uploading computer, which may be an individual computer where the user is performing direct file upload, or the Gateway server. This local anonymisation ensures that patient information does not leave the clinical institution.

**Fig. 2:**
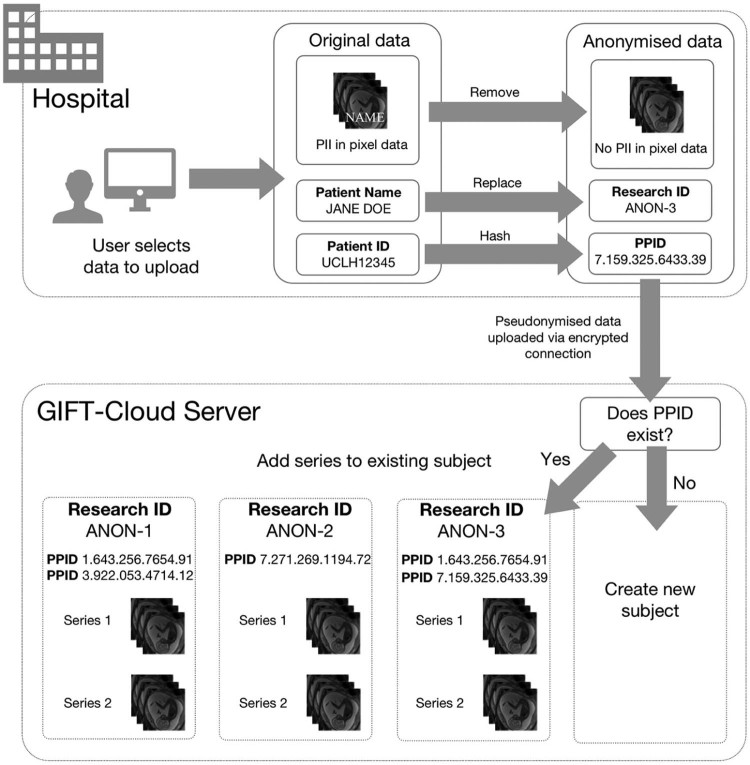
Diagram showing the process for anonymisation and subject matching during upload of imaging data to GIFT-Cloud. Anonymisation is performed within the clinical institution; if uploaded via PACS this is performed by the Gateway server, while if the user uploads data directly from their computer then this anonymisation is performed on their local computer. Before uploading, the image metadata are examined to determine the type of anonymisation required. The Patient ID is translated into a pseudonymised patient identifier (PPID). This PPID is used to determine if the subject already exists on the server and to obtain the pseudonymous Research ID for that subject. If no subject exists, then a new subject is created with a new pseudonymous Research ID. Within the metadata, the Patient ID is replaced by the PPID and the patient name is replaced by the Research ID. Required data grouping fields such as the Series Instance UID are replaced with a SHA-1 hash of their value.

The DICOM metadata anonymisation procedure is shown in Fig. 2. Fields required for correctly grouping data, such as the patient identifier, series instance UID and study instance UID, are pseudonymised using a one-way SHA-1 hash algorithm. This produces a consistent identifier for each subject and series, ensuring data can always be grouped correctly, even if uploaded at different times, while preserving pseudo-anonymity (see Fig. 2). Patient names are replaced by an arbitrary human-readable pseudonymous identifier (Research ID). Additional fields documented in the DICOM standard that may contain information that could help to identify the patient, such as date of birth or accession number, are removed. The anonymisation of other fields is configurable per-project using scripts written in the DicomEdit language [19]. All private (vendor-specific) fields, which are undocumented and sometimes contain patient information, are removed unless specifically whitelisted in the anonymisation script. As an additional security measure, GIFT-Cloud Uploader verifies before each upload that fundamental identifying fields (patient name, patient ID, date of birth and accession number) have been modified, in order to prevent upload in the case of a misconfigured or missing anonymisation script.

Some types of medical images contain patient information in annotations burnt into the pixel data. This is often the case for 2D and 2D+t ultrasound images, and can in some cases be true for MR localisers and scout images. This is a remnant of paper and film based organisation, which for those types of applications is still widespread. These annotations are vendor specific, but for a given scanner model, modality and software version, their location is generally consistent. The DICOM standard contains a field to indicate the presence of burnt in annotations but this is optional and cannot be relied upon to signal the presence and location of annotations [20].

**Fig. 3:**
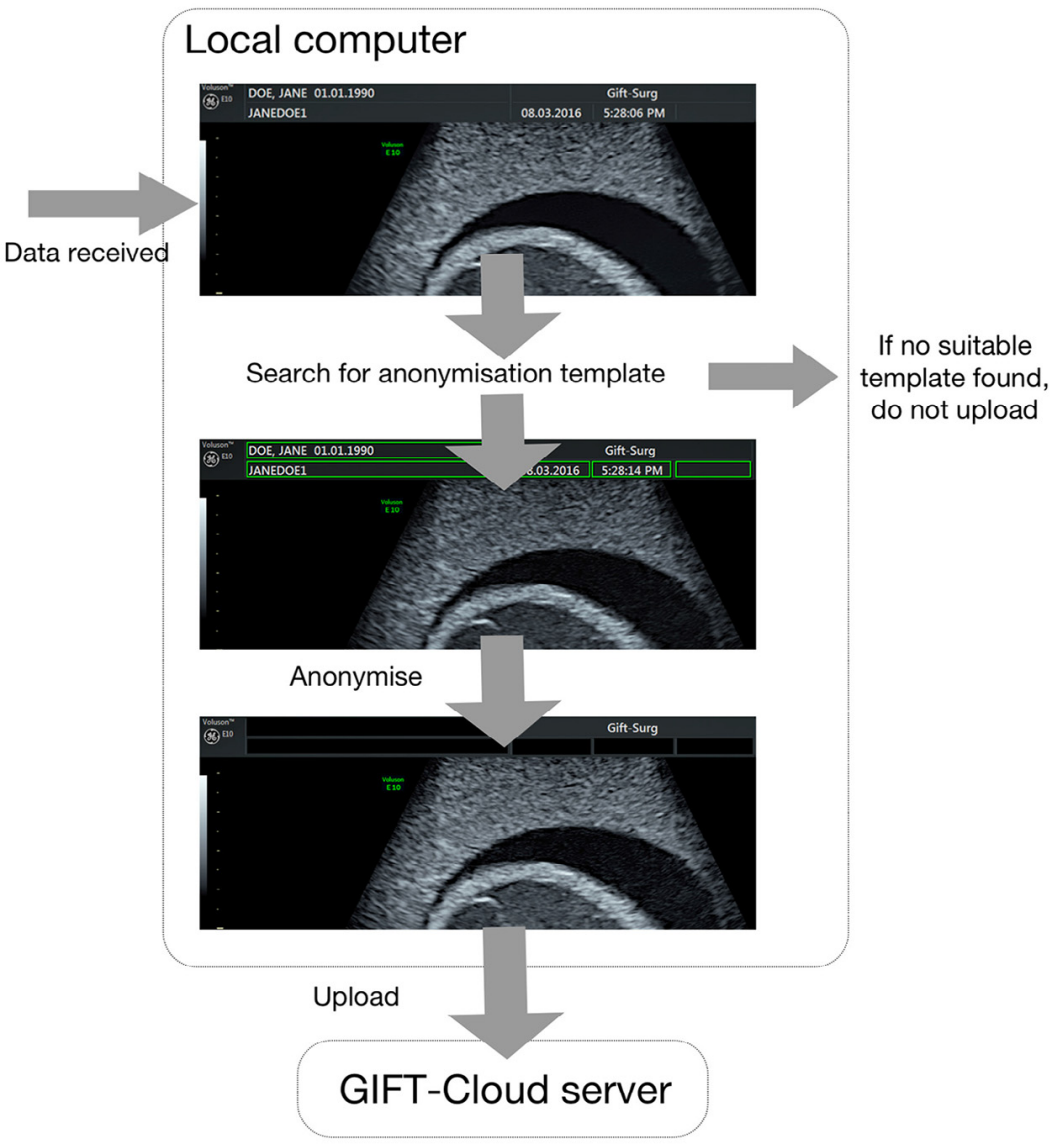
Illustration of automated pixel data anonymisation procedure applied before uploading images. The image shown is a cropped ultrasound scan of a training phantom. The image metadata are examined to determine if pixel data anonymisation is required. If so, a suitable template for the scanner model and image resolution is located that specifies which regions of the image contain personal identifiable data. These regions are blacked out before uploading. If no suitable template is found, the data are not uploaded.

Automated pixel data anonymisation is achieved using a template database (see Fig. 3). The Uploader uses DICOM information describing the source and type of image, including the scanner model, scanner software version, image modality and resolution, to find the template defining which regions must be blacked out in each image. The black-out procedure is performed automatically on each image or video frame using software extended from DicomCleaner [9]. If the DICOM standard permits an image type to contain burnt-in annotations, but no suitable template is found, then the data are not uploaded. An interactive tool enables users to create new templates defining the regions to black out on the images from scanners that are not yet in the template database.

### 3.6 Storing pseudonymous patient identifiers

Some research projects require clinicians to maintain a list mapping patient identifiers to the pseudonymous Research IDs used in the research data. This could for example be used in longitudinal follow-ups with patients. The Gateway installed within each institution maintains this mapping in a local password protected file that does not leave the hospital network.

### 3.7 Data grouping

For multi-modal or time series data analysis, it is necessary that data are grouped under the same subject label, even if those data were uploaded at different times. However, typical anonymisation processes destroy the identifiers that make such grouping possible. GIFT-Cloud solves this situation by using the pseudonymised patient, study and series identifiers to automatically match the data to the existing subjects and series. Because this mechanism is independent of the Gateway or local computer that performs the uploading, it ensures correct grouping of data regardless of how the data are uploaded, even if they are transferred from different institutions.

### 3.8 Data security and access control

The GIFT-Cloud server does not receive nor store any personal identifiable data, only pseudo-anonymised data. All data exchange over the Internet is encrypted and a server certificate ensures clients can only connect to the genuine server. Firewalls on the GIFT-Cloud Server and Gateways restrict access only to trusted clients.

**Fig. 4:**
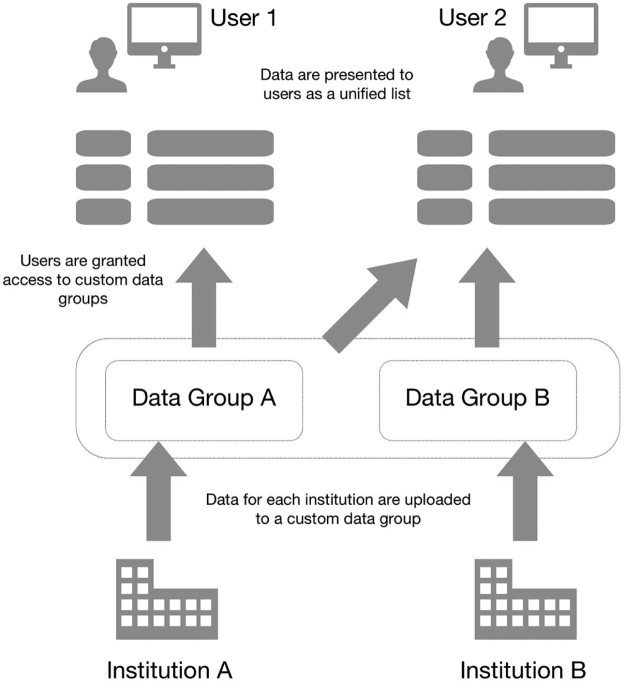
The data access permissions model for GIFT-Cloud. In this example, the current data sharing agreements between institutions permit User 1 to access data from Institution A but not from Institution B. User 2 is permitted to access data from both institutions.

Data access requires a personal account. GIFT-Cloud stores data from each institution in a separate data group (see Fig. 4) and access to these groups can be individually configured for each account. This supports adherence to data-sharing agreements that may vary between institutions.

## 4 Case study: placental segmentation

To illustrate the integration of GIFT-Cloud with clinical workflow, research and development, we describe an application in fetal surgical planning and image-guided surgery that we are currently developing as part of the GIFT-Surg project.

### 4.1 Background

Identical twin placental abnormalities such as twin-to-twin transfusion syndrome (TTTS) [21] and selective intrauterine growth restriction (sIUGR) [22] can have a major impact on maternal and fetal outcome. Both conditions can be treated effectively using minimally invasive fetoscopic surgery. Advanced image-based surgical planning techniques have the potential to improve efficacy and reduce treatment-related morbidity and mortality [23]. However, such an image-based diagnosis and surgical planning system requires robust and accurate segmentation of fetal and maternal organs from MRI images acquired during the diagnostic phase. To assist with this, we have developed an advanced computational method for semi-automated organ segmentation, focussing initially on the placenta [24]. The ongoing development and assessment of this method requires recurring testing and validation with new data.

### 4.2 Data

Retrospective and prospective fetal MRI scans are obtained from UCLH and UZ Leuven as part of routine scanning, with approval by the ethical committees and maternal consent for research use. For this research, datasets are selected that show complete placenta volumes for the second trimester.

### 4.3 Workflow

**Fig. 5:**
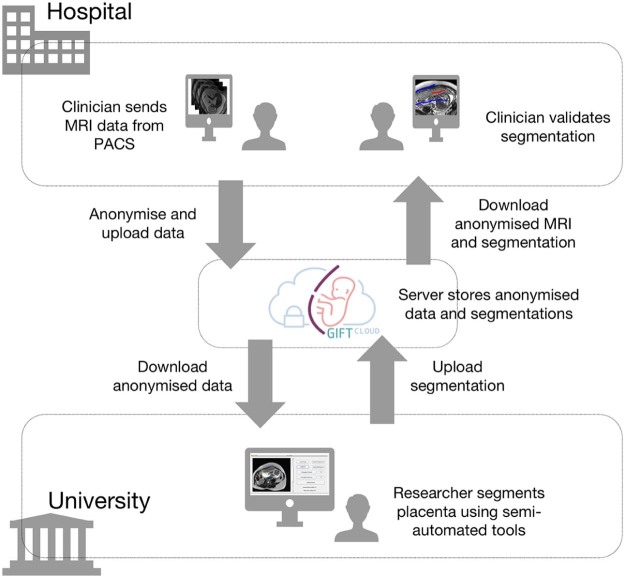
Data workflow showing how GIFT-Cloud is used in the development of an algorithm for placental segmentation for fetal surgery applications. The clinician uses the PACS to send a suitable dataset to GIFT-Cloud. The data are sent by DICOM Push to the GIFT-Cloud Gateway, which anonymises, encrypts and uploads the data to the GIFT-Cloud server. The researcher can then download the anonymised data, perform the segmentation and upload the resulting segmentation mask, which will be grouped in the same subject as the anonymised data. The clinician can now download and compare the anonymised data and segmentation for validation. Through such workflows GIFT-Cloud makes it possible to easily share new results during the active development stages of novel imaging methods.

The workflow is illustrated in Fig. 5. The clinician selects datasets using the hospital PACS or the medical record, and sends them to the GIFT-Cloud Gateway node. The clinicians need not be concerned about anonymisation or upload procedure which happens automatically. The hospital Gateway internally stores the mapping between patient ID and pseudonymised research ID for future reference. Researchers retrieve the anonymised data using software integrated with GIFT-Cloud, perform the segmentation using Slic-Seg [24] and upload the resulting segmentation mask to the server. The clinicians can access and evaluate the resulting segmentation through the GIFT-Cloud web interface or by downloading the data to their own systems.

## 5 Evaluation

### 5.1 Feature summary

Table 2 summarises key features available to end users of GIFT-Cloud. These include: standard features provided by XNAT; user-facing features implemented by GIFT-Cloud using XNAT's programmatic REST API; and novel features developed for GIFT-Cloud.

### 5.2 Capacity

Data storage does not present a fundamental limitation for this work as storage is managed by a PostgreSQL database on an extensible hard disk. GIFT-Cloud is designed with an expected need for storing 20,000 imaging series from 1000 subjects, comprising up to one million images in total. We expect the operating limits of the system to be exponentially higher than this, based on XNAT usage by other projects. For example, the Vanderbilt University IIS CCI database, based on XNAT, hosts more than a quarter of a million scans for over 28,000 subjects [25].

### 5.3 Scalability

The system allows the practical addition of unlimited new clinical sites by installing local Gateways, with no limit to the number of users who can access the system. The total capacity of the system is limited by the single server design, which is adequate for our purposes. However, if individual sites regularly require large volumes of data upload or download, a redundant server design may be more appropriate.

### 5.4 Performance

GIFT-Cloud is designed to prioritise a low impact on network infrastructure over speed and latency; therefore data upload is bandwidth-limited in a configurable manner. Speed of upload is therefore not a consideration for this work.

### 5.5 Fault tolerance

The Gateway servers asynchronously store and upload data received from the PACS. To prevent data loss, files are physically stored on the Gateway servers until notified of successful storage by the Server. If the Server connection is not available or data upload fails, uploading is re-attempted multiple times with an exponentially increasing delay.

### 5.6 Testing

The Server and Uploader software include a suite of automated unit, integration and system tests. After installing the Gateway systems at each hospital, the anonymisation mechanisms were tested with dummy data to ensure compliance with data anonymisation policies.

### 5.7 Data governance

GIFT-Cloud fulfils the requirements of patient confidentiality and information governance processes within all the collaborating institutions and in the UK were passed by the appropriate NHS Caldicott guardian and were cleared by the London Hampstead Research Ethics Committee (15/LO/1488).

## 6 Discussion and conclusion

GIFT-Cloud provides a secure, open-source platform for two-way sharing of research data across multiple institutions [5]. GIFT-Cloud is designed to meet the demands of collaborative research projects by simplifying data transfer and automating anonymisation processes.

GIFT-Cloud fulfils the following key requirements: firstly, the ability to easily integrate with local IT infrastructure of the institutions that provided clinical data and expertise, with the end user's systems such as PACS or electronic medical record, and within routine clinical workflow. Secondly, the support for varied collaboration agreements between institutions and related access control restrictions. Thirdly, the support not only for DICOM data but also for including images and video available in other commonly used formats.

Ongoing work will identify and integrate support for additional anonymisation approaches that may be needed for future data protocols and modalities. Other planned improvements include extending support for additional data formats and video codecs, and adding a configurable uploading bandwidth limitation to reduce the impact of the Gateway servers on the PACS systems when dealing with large data streams. An improved configuration updating system will allow new modalities and pixel data anonymisation templates to be added via the server without requiring software updates to the Gateway and user software.

GIFT-Cloud will promote interdisciplinary research within the GIFT-Surg project, facilitating the development of new software for fetal surgical planning and image-guided surgery. The automated processes significantly reduce the time burden on clinicians and researchers in data anonymisation and transfer, encouraging more data sharing. Researchers benefit from a centralised repository of all available data with secure access and automated backup. Research software can be written to automatically fetch the data directly from the server, instead of requiring the developer to manually copy data to their machine. Additionally, the ability to share segmentations and other analysis results back to the GIFT-Cloud Server allows clinical users to access and evaluate these results.

These features are useful not only to GIFT-Surg; they can more generally benefit medical imaging research projects where data sharing is required between researchers and with clinical institutions. The supporting XNAT framework further provides a wide range of customisations and extensible features. By building on cross-platform architecture and publishing our software under an open-source licence, we offer this system for improving collaboration across medical imaging research.

## Funding

This work was supported through an Innovative Engineering for Health award by the Wellcome Trust [WT101957], the Engineering and Physical Sciences Research Council (EPSRC) [NS/A000027/1] and a National Institute for Health Research Biomedical Research Centre UCLH/UCL High Impact Initiative. Jan Deprest is funded by the Fonds voor Wetenschappelijk Onderzoek Vlaanderen (FWO) [1.8.012.07] and Great Ormond Street Hospital Children's Charity. Anna L. David is supported at UCL/UCLH by funding from the Department of Health NIHR Biomedical Research Centres funding scheme. Sébastien Ourselin receives funding from the EPSRC [EP/H046410/1, EP/J020990/1] and the MRC [MR/J01107X/1].

## Conflicts of interest

None.

## Figures and Tables

**Table 1:** System requirements. This table describes the core hardware and software requirements for the components of the GIFT-Cloud system, and the current versions used in the GIFT-Cloud system installed at UCL.

	Minimum requirement	UCL system
*GIFT-Cloud Server*		
Operating system	Linux/Windows/macOS	CentOS Linux 7.2
PostgreSQL	9.1	9.2
Oracle Java SDK	1.7	1.7
Apache Tomcat	7.0	7.0
XNAT	1.6	1.6
*GIFT-Cloud Gateway*		
Operating system	Linux/Windows/macOS	Microsoft Windows Server 2008 R2
Oracle Java JRE	1.7	1.8
Java Advanced Imaging	1.1	1.1
GIFT-Cloud Uploader	1.1.10	1.1.10

**Table 2:** Summary of GIFT-Cloud features including those provided by the XNAT system. GIFT-Cloud extends XNAT with additional tools and novel features. XNAT features labelled “via API” are not part of the standard XMAT 1.6 user interface but are available programmatically through the REST API and may be invoked by third-party tools such as those provided by GIFT-Cloud.

Feature	XNAT 1.6	GIFT-Cloud
Web user interface	✓	✓
REST API	✓	✓
Automatic subject grouping by PPID lookup		✓
Interactive single-subject data upload	✓	✓
Automated multi-subject data upload	via API	✓
Remote DICOM Gateway for query/retrieve	✓	✓
Remote DICOM Gateway for anonymisation and store		✓
PACS integration	via API	✓
PACS integration on-site anonymisation and encryption		✓
Configurable client-side metadata anonymisation	✓	✓
Configurable client-side pixel data anonymisation		✓
DICOM CT, MR, US support	✓	✓
NIFTI, Analyze support	via API	✓
Automatic MPEG to DICOM video conversion		✓
